# A novel diagnostic method for a rare fungus: FcMBL facilitates *Wickerhamomyces anomalus* identification in an immunocompromised neonate

**DOI:** 10.1016/j.mmcr.2023.100614

**Published:** 2023-10-21

**Authors:** Conor J. Higgins, Kerry-Anne Kite, Nigel Klein, Michael Super, Michael T. McCurdy, Darren Hargrave

**Affiliations:** a The Robert Larner, M.D. College of Medicine at the University of Vermont, Burlington, VT, USA; b Great Ormond Street Institute of Child Health, London, United Kingdom; c Great Ormond Street Hospital, London, United Kingdom; d Wyss Institute for Biologically Inspired Engineering, Harvard University, Boston, MA, USA; e University of Maryland School of Medicine, Baltimore, MD, USA; f BOA Biomedical Inc., Cambridge, MA, USA

**Keywords:** *Wickerhamomyces anomalus*, FcMBL, Fungemia, MALDI-TOF MS, Neonate, Case report

## Abstract

Fungemia negatively impacts patient outcomes, current diagnostics lack sensitivity to identify emerging rare mycoses, and fungal infections are increasing in prevalence, variety, and resistance. We report a case of *Wickerhamomyces anomalus* in an immunocompromised neonate in which FcMBL bead-based matrix-assisted laser desorption/ionization time-of-flight (MALDI-TOF) mass spectrometry (MS) resulted in species identification roughly 30 hours before standard pathogen identification methods. Deploying FcMBL bead-based MALDI-TOF MS may improve the speed and accuracy of identification, and therefore treatment, of rare pathogens.

## 1 Case report

### 1.1 Background

**Fig. 1:**
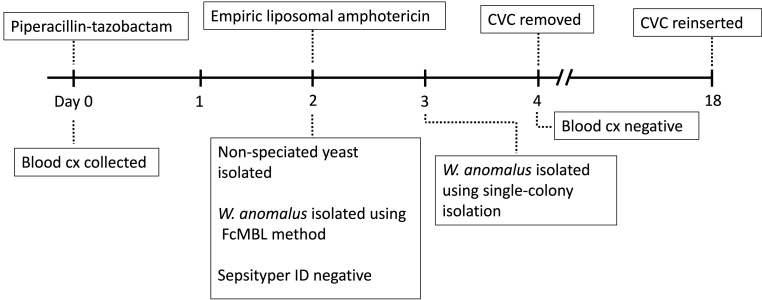
Timeline of the patient's blood culture history and associated treatments. Day 0 indicates the first day of suspected infection.

**Fig. 2:**
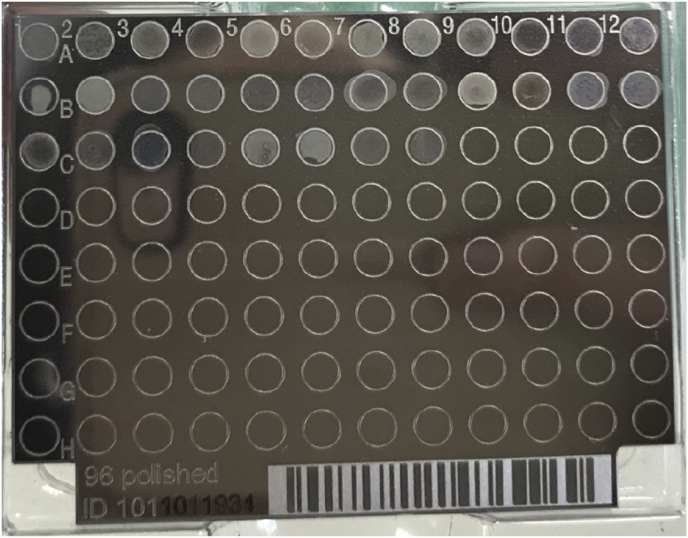
Photograph of the mass spectrometry plate where samples are plated after either Bruker Sepsityper**®** or FcMBL-based processing but prior to MALDI-TOF analysis.

**Fig. 3:**
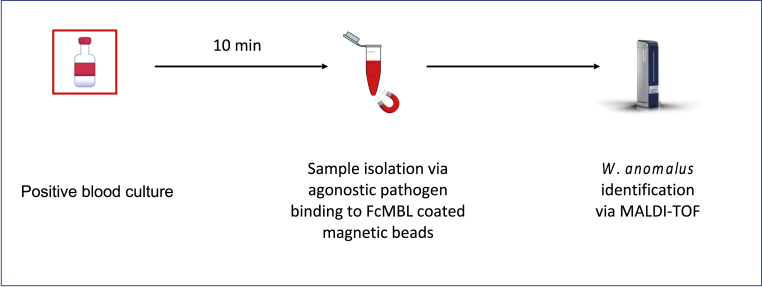
Schematic of pathogen isolation via FcMBL magnetic beads. Agnostic binding facilitates rapid pathogen concentration following a positive blood culture. The sample is then eluted off FcMBL beads and processed via MALDI-TOF.

**Fig. 4:**
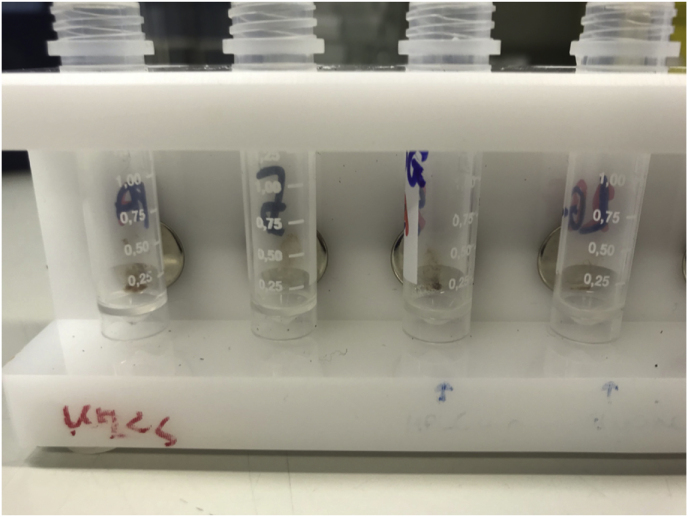
After agnostic pathogen binding, FcMBL beads are isolated via a magnetic rack and eluted for pathogen analysis.

Fungemia, an escalating global problem in part due to increased incidence and rising antimicrobial resistance (AMR), negatively impacts patient outcomes and taxes healthcare resources. Up to half of all episodes of candidemia occur in the intensive care unit (ICU), with costs ranging from US$35,000 to US$68,000 for each instance above expected ICU costs for a critically ill patient without candidemia [1,2]. Moreover, the emergence and adverse clinical consequences of rare and invasive fungal species have increased due to a plethora of reasons that include AMR, increased antibiotic exposure, and central venous catheter use [3,4]. Unfortunately, this trend is expected to continue. A recent review of fungal bloodstream infections in a pediatric intensive care unit determined that non-*Candida spp*. comprised over 61% of all instances of fungemia [3]. Unfortunately, fungal identification is often delayed due to low clinical suspicion and microbiological diagnostic limitations. Negative test results cannot exclude the diagnosis of infection in an immunocompromised host, largely due to the slow rate of microbiological growth and lack of test sensitivity [5]. Such diagnostic delays result in patient harm, and novel solutions are warranted to improve the speed and accuracy of fungal diagnosis (see Fig. 1, Fig. 2, Fig. 3, Fig. 4).

One such yeast, *Wickerhamomyces anomalus*, has previously been isolated in neonates and otherwise immunosuppressed individuals on corticosteroids or chemotherapy [3,6]. *W. anomalus* can be found in organic matter including fruits, plants, and oils. However, other than its first report as an isolate from a child's lungs in 1958 and an outbreak in a neonatal ICU in 1996, relatively few published reports of *W*. *anomalus* exist [7].

Rising rates of fungemia are particularly worrisome due to conventional methods delaying timely detection and preventing appropriate treatment. Conventional methods of pathogen detection struggle to correctly differentiate fungal genera and species. Even more advanced molecular diagnostics like PCR have limitations in discriminating among species, as demonstrated by one study highlighting the superiority of matrix-assisted laser desorption/ionization time-of-flight mass spectrometry (MALDI-TOF MS) to differentiate among several yeast species, such as *W. anomalus* [8].

Despite its historically favorable susceptibility pattern, several instances of antifungal resistance in *W. anomalus* have been reported [9]. Resistance to voriconazole was described in an outbreak in a Brazilian pediatric ICU, and a separate study found 8 of 15 strains to be fluconazole-resistant while 6 of 8 strains were resistant to itraconazole and ketoconazole *in vitro* [6,9]. The clinical impact of not accurately identifying an infecting organism in a timely fashion is significant, as both overtreating and undertreating invading pathogens adversely affect patient outcomes. For example, every hour adequate antimicrobial administration is delayed in patients with septic shock, survival decreases by 7.6% [10]. To avoid undertreating an infection, empiric broad-spectrum antibiotics are routinely administered in patients presenting with sepsis. Unfortunately, unnecessary antimicrobials increase patients’ vulnerability to clinical complications and fuel growing rates of hospital and global AMR. These problems with both overtreatment and undertreatment of infections can be mitigated with accurate and rapid pathogen identification.

Early clinical suspicion and improved methods to identify fungemia help to ensure proper antimicrobial use, as inappropriate and unnecessary empiric antibiotics are associated with higher mortality [10]. Rapid detection becomes even more necessary in immunocompromised and neonate populations who may not exhibit more common manifestations of immune dysfunction.

Mannose binding-lectin (MBL), produced by the liver, functions as an opsonin and plays a key role in the innate immune system and immediate defense against harmful pathogens [11]. MBL broadly binds a wide range of pathogens and their associated debris [12]. The engineered FcMBL molecule consists of the carbohydrate-identifying region of MBL fused to the fragment crystallizable (Fc) region of immunoglobulin G. FcMBL demonstrates similar capabilities as the natural opsonin MBL, successfully binding to over 195 pathogen isolates from over 100 Gram-positive and Gram-negative bacteria, viruses, parasites, and fungi [12]. FcMBL conserves binding activity of MBL, while regions responsible for coagulation and complementation activation are removed. The result is an engineered protein with the unique ability to capture and concentrate pathogens without triggering an immune response. FcMBL can be conjugated to virtually any form factor, including the diagnostic beads further described in this case.

We report a case of *W. anomalus* detection in an immunocompromised neonate using FcMBL magnetic bead-based MALDI-TOF MS at the Great Ormond Street Hospital (GOSH) in London, UK. This novel technology resulted in the capture, concentration, and identification of *W. anomalus* 30 hours before methods used in standard clinical practice. Deploying FcMBL-based MALDI-TOF MS to identify pathogens may improve the identification and treatment of rare and fastidious infectious organisms.

### 1.2 Clinical case

A 30-month-old boy being treated for an atypical teratoid rhabdoid tumor (ATRT), a relapsed aggressive brain tumor, who previously underwent an autologous transplant with peripheral stem cell rescue one year earlier, was admitted for inpatient multi-agent chemotherapy via an indwelling double-lumen central venous catheter (CVC). During his admission, he experienced rigors and his temperature was found to be 38.5 °C. He exhibited no focal signs of an infectious source. At that time (Day 0), his laboratory data revealed 2.16 × 10^9^ white blood cells per liter (L) with 1.01 × 10^9^ neutrophils per L, and a C-reactive protein <5 mg (mg) per L. Following acquisition of blood (from both CVC lumens), stool, and urine cultures, intravenous piperacillin-tazobactam was initiated. On Day 2 of ongoing fevers, the microbiology team isolated a nonspeciated yeast from the blood culture from both CVC lumens, and 3 mg/kg per day liposomal amphotericin was promptly initiated. On Day 3, a repeat blood culture sample became positive for *W. anomalus,* identified via single colony-isolation from a subculture. The evaluation for the presence of a disseminated fungal infection (i.e., abdominal ultrasound, echocardiogram, retinal fundoscopy) was negative. Both collected on Day 2, galactomannan and beta-D-glucan were negative at an external lab using 1 mL clotted serum, and 18s rDNA screening PCR was negative on-site at GOSH. These samples were collected at the time of the second positive blood culture. On Day 4, the CVC was removed, a peripheral cannula was inserted, and 3 mg/kg liposomal amphotericin was continued for 14 days prior to inserting a new CVC. Following CVC removal, his fever resolved and further cultures were negative. The patient made a complete recovery from his CVC-associated fungemia and resumed the planned intensive multi-agent chemotherapy.

Blood culture identification requires 1–5 days of incubation to grow enough pathogens to be identified using most molecular diagnostics. In this case, 10 mL (mL) of blood was removed via the patient's CVC and cultured in Bactec™ bottles in an incubator. The anaerobic bottle flagged first and subsequently underwent identification using existing pathogen diagnostic protocols at GOSH with Bruker's Sepsityper® kit (Bruker Daltonics, Bremen, Germany). The Sepsityper® utilizes centrifugation to separate the supernatant of positive blood cultures to isolate pathogens for subsequent identification using MALDI-TOF MS.

### 1.3 FcMBL processing

The clinical research team at GOSH concomitantly deployed an experimental protocol exploring the capabilities of the magnetic bead-bound FcMBL molecule to enhance pathogen capture in cultured samples. Although typically only used for research and not clinical purposes, the team implemented the FcMBL-based protocol, which utilizes FcMBL-coated magnetic beads to enhance pathogen isolation and concentration.

To perform the FcMBL-based method, 1 mL supernatant from the positive blood culture bottle was incubated with 100 μL of 5% saponin (Millipore) and 100 μL (5 μg) of FcMBL beads with 10 mmol (mM) glucose and tris buffered saline with Tween (TSBT) 5 mM calcium (Ca^2+^) at 22 °C for 5 minutes in 2 mL Eppendorf tubes. The tubes were then placed in a magnetic holder for 1 minute to separate the pathogen-bound FcMBL magnetic beads from the supernatant. The supernatant was removed via a pipette and the tubes placed in a rack for two cycles of washing with 1 mL of HBSS and calcium. The pathogen was then eluted off the FcMBL beads in 20 μL formic acid and 20 μL acetonitrile [13]. 1 μL of supernatant was layered onto the target plate. Time to identification via MALDI-TOF MS was approximately 9 minutes. Although the Bruker Sepsityper**®** failed to accurately identify the yeast *W. anomalus,* the FcMBL processing method successfully did.

## 2 Discussion

Methods to expedite pathogen identification are sorely needed for timely targeted intervention in instances of fungemia and sepsis. By separating microbes from the blood using host cell lysis and centrifugation, the Sepsityper® kit can detect Gram-negative bacteria with rates ranging from 93 to 98%, but scores drop considerably when detecting Gram-positive bacteria and yeast, with scores of 58–80% and 57–82%, respectively [14]. Although generally a highly accurate test, the Sepsityper® has certain limitations that enable FcMBL-based capture to augment the pathogen diagnostic capabilities of molecular tests.

Although the Sepsityper® and FcMBL magnetic bead-based protocols require similar amounts of time to perform, the Sepsityper® method was initially unable to identify *W. anomalus* via MALDI-TOF. The GOSH clinical laboratory then cultured the yeast for an additional 30 hours before standard-of-care single-colony detection by MALDI-TOF MS. The FcMBL bead-based processing method, however, captured and concentrated a sufficient amount of *W. anomalus* to permit immediate MALDI-TOF MS identification, resulting in accurate identification roughly 30 hours before identification using the Sepsityper® method.

Unfortunately, because the FcMBL protocol was only performed under research conditions after lack of identification by the Sepsityper® kit, no clinical action could be taken based on the FcMBL bead-based identification of *W. anomalus*. To test the experimental FcMBL bead-based pathogen binding in a clinical setting, this protocol was experimentally deployed concurrently with the standard clinical protocol at GOSH. Fortunately, the GOSH clinical team empirically initiated the broad-spectrum antifungal liposomal amphotericin, which did not require adjustment 30 hours following successful identification of the fluconazole- and micafungin-resistant fungus via single-colony isolation.

Fungemia presents a unique challenge in the field of pathogen identification. Though *C. albicans* historically accounted for almost 80% of nosocomial candidemia, it now contributes to less than half of all inpatient instances [15]. This trend is not isolated to just *Candida* species, as rates of invasive and rare fungal infections continue to rise [3,4]. The increasing incidence can at least partially be attributed to organ transplantation, broad-spectrum antibiotics, and long-term venous access [16]. Of concern, fungemia is associated with both short-term and long-term mortality, with one study citing 1-month mortality rates of 42% and 4-year mortality rates of 86% [17]. An immediate need exists for expedited pathogen identification to allow for targeted antimicrobial treatment.

The FcMBL bead-based protocol is a novel technology that enables effective capture and concentration of pathogens from the positive blood culture bottle. This protocol has previously demonstrated 100% sensitivity for clinical candidemia compared to 33% sensitivity for Sepsityper® [13]. Moreover, the FcMBL method identified 100% of clinical *Candida* to the genus level in the same clinical study, whereas the Sepsityper® kit identified only 70.8% of the samples to the genus level. Implementing the FcMBL method into the MALDI-TOF MS pathogen diagnostic clinical protocol may facilitate more rapid pathogen identification and appropriate antimicrobial therapy. Although the clinical utility of FcMBL has been demonstrated for pathogen identification in urine, aqueous humor, and wound swab and fluids [[18], [19], [20]]. However, further studies involving larger cohorts are required before implementing into standard practice.

## Funding

This research did not receive any specific grant from funding agencies in the public, commercial, or not-for-profit sectors.

## Consent and ethics approval

Written informed consent was obtained from the patient for publication of this case report and accompanying images. A copy of the written consent is available for review by the Editor-in-Chief of this journal on request.
